# Supplementary material for: Evaluation of nutritional supplements prescribed, its associated cost and patients knowledge, attitude and practice towards nutraceuticals: A hospital based cross-sectional study in Kavrepalanchok, Nepal
Source: PLoS One. 2021 Jun 4;16(6):e0252538. doi: 10.1371/journal.pone.0252538 (PMC8177421; doi:10.1371/journal.pone.0252538)
Supplement: S1 File — KAP questionnaire. (DOCX) [file pone.0252538.s001.docx]

**APPENDICE A: RESEARCH QUESTIONNAIRE**

**Direction:** Please attempt questions by tick ( ) mark to the options provided with each question or by supplying the answer if space is provided. Some of the questions may have multiple answer.

Patient Visit On:

Medical Surgical Orthopedic Obstetrics & Gynecology

**Part I: Socio-demographic information**

1. Age ……………Years

15-29 30-44 45-59 Above 60

1. Gender

Male Female Others (Specify)…..

1. Ethnicity:

Brahmin Chettri Newar Others (Specify)…..

1. Religion:

Hindu Buddhist Christian Others (specify)

1. Educational status:

Illiterate Literate

*If literate which level*

Just read and write Primary Secondary Higher secondary & above

1. Marital status

Married Unmarried Divorced Others (specify)

1. Occupation

Housewife Service Business Others (specify)

**Part II: Question Related to practices of Nutraceutical**

1. Have you ever consume nutraceutical?

Yes No

1. How often do you consume nutraceutical?

Occasionally 3-5 times a week Daily Not Sure

1. What type of supplements do you consume? (Multiple Response)

Multivitamins Vitamin E Vitamin B complex Vitamin C

Folic acid Iron Calcium Protein Powder

Herbal supplements Others (specify)

1. Do you think counseling is important before consuming nutraceutical?

Yes No Do not know

1. Do you consult professional medical help when taking nutraceutical?

Yes No Do not know

1. What is the source of information? (Multiple Response)

Health personnel Friends Multimedia (TV, Radio, Internet, etc.)

Books Others

1. Is information available are adequate?

Yes No Do not know

1. How do you buy Nutraceutical?

With prescription Without prescription

1. From where did you buy nutraceutical?

Hospital Pharmacy Community Pharmacy Department Store

Others (specify)………..

**Part III: Question related to knowledge of nutraceutical**

1. Do you know what Nutraceutical are?

Yes No

1. Do you think use of nutraceutical is always safe?

Yes No Do not know

1. Do you think that taking a drug, food or drinks with nutraceutical might interact with each other?

Yes No Do not know

1. What is the reason for consuming nutraceutical? (Multiple Response)

Treatment of any disease Maintain good health

Ensure adequate nutrition Weight Loss

Enhance appearance Meet increased energy needs

Prevent disease No specific reason

**Part IV: Question related to Attitude towards nutraceutical**

1. Nutraceutical are needed if a person feels tired and rundown.

Strongly disagree Disagree Neutral Agree Strongly Agree

1. Nutraceutical make one feel better physically.

Strongly disagree Disagree Neutral Agree Strongly Agree

1. Nutraceutical usually improve a person’s appearance.

Strongly disagree Disagree Neutral Agree Strongly Agree

1. Body fat can be lost by taking certain type of nutraceutical.

Strongly disagree Disagree Neutral Agree Strongly Agree

1. One cap skip meals and just take nutraceutical.

Strongly disagree Disagree Neutral Agree Strongly Agree

1. The nutrients supplied by food need to be supplemented.

Strongly disagree Disagree Neutral Agree Strongly Agree

1. Nutraceutical is necessary for all ages.

Strongly disagree Disagree Neutral Agree Strongly Agree

1. Nutraceutical is generally harmless.

Strongly disagree Disagree Neutral Agree Strongly Agree

1. Regular use of supplements prevent chronic diseases.

Strongly disagree Disagree Neutral Agree Strongly Agree

1. Nutraceutical can prevent cancers.

Strongly disagree Disagree Neutral Agree Strongly Agree

1. Health professional should promote use of supplements.

Strongly disagree Disagree Neutral Agree Strongly Agree

1. Nutraceutical should be sold only on prescription of a registered medical practitioner.

Strongly disagree Disagree Neutral Agree Strongly Agree

1. Manufacture and sale of nutraceutical should be monitored by a regulatory body.

Strongly disagree Disagree Neutral Agree Strongly Agree

1. Use of nutraceutical are just waste of money.

Strongly disagree Disagree Neutral Agree Strongly Agree

**Thank you for your time and patience!**
