# Supplementary material for: Evaluation of nutritional supplements prescribed, its associated cost and patients knowledge, attitude and practice towards nutraceuticals: A hospital based cross-sectional study in Kavrepalanchok, Nepal
Source: PLoS One. 2021 Jun 4;16(6):e0252538. doi: 10.1371/journal.pone.0252538 (PMC8177421; doi:10.1371/journal.pone.0252538)
Supplement: Attachment — Submitted filename: Response Sheet.docx [file pone.0252538.s004.docx]

**February 16, 2021**

**Point to point response to the reviewers’ comments**

We are very grateful for the reviews provided by the editors and each of the external reviewers of this manuscript. The comments are encouraging and the reviewers appear to share our judgment that this study and its results are clinically important. Please kindly see below, in blue, our detailed response to comments.

All page numbers and line numbers refer to the manuscript file with tracked changes.

Sweta Shrestha and team.

**Authors Response to Reviewers**

**Comments to the Author**

1. Is the manuscript technically sound, and do the data support the conclusions?

The manuscript must describe a technically sound piece of scientific research with data that supports the conclusions. Experiments must have been conducted rigorously, with appropriate controls, replication, and sample sizes. The conclusions must be drawn appropriately based on the data presented.

Reviewer #1: Partly

Reviewer #2: No

**Author’s Response:**We have revised the manuscript according to the reviewer’s comments.

2. Has the statistical analysis been performed appropriately and rigorously?

 Reviewer #1: I Don't Know

Reviewer #2: I Don't Know

**Author’s Response:** We again revised the statistical analysis.

3. Have the authors made all data underlying the findings in their manuscript fully available?

The [PLOS Data policy](http://www.plosone.org/static/policies.action#sharing) requires authors to make all data underlying the findings described in their manuscript fully available without restriction, with rare exception (please refer to the Data Availability Statement in the manuscript PDF file). The data should be provided as part of the manuscript or its supporting information, or deposited to a public repository. For example, in addition to summary statistics, the data points behind means, medians and variance measures should be available. If there are restrictions on publicly sharing data—e.g. participant privacy or use of data from a third party—those must be specified.

Reviewer #1: Yes

Reviewer #2: No

**Author’s Response:**

All the available data on socio-demographic variables, individual knowledge score, attitude and practice scores have been made available in an excel sheet as well as in SPSS. Data on the total number of nutraceuticals prescribed per prescription, frequency of types of nutraceutical prescribed and nutraceutical cost per patient has also been provided. Besides, ethical approval and questionnaire in English have also been made available.

4. Is the manuscript presented in an intelligible fashion and written in standard English?
PLOS ONE does not copyedit accepted manuscripts, so the language in submitted articles must be clear, correct, and unambiguous. Any typographical or grammatical errors should be corrected at revision, so please note any specific errors here.

 Reviewer #1: No

Reviewer #2: No

**Author’s Response:** We have revised the manuscript. We have also revised typographical and grammatical errors.

5. Review Comments to the Author

Please use the space provided to explain your answers to the questions above. You may also include additional comments for the author, including concerns about dual publication, research ethics, or publication ethics. (Please upload your review as an attachment if it exceeds 20,000 characters)

Reviewer #1:

Title: Title statement (“……visiting OPD in Nepal and”) seems strange it should rather be “..Hospital OPD in Nepal” . As categorizations/ operational definition of nutraceutical wasn't clear (see below comments in method section ), "More Precisely, I suggest the title something like “Evaluation of Nutritional supplements prescribed, its associated Cost and Patients knowledge, attitude and practice towards Nutraceuticals: A hospital based cross-sectional study in Kabrepalanchok, Nepal”

**Author’s Response:** The title has been revised as per the suggestion of the reviewer. The revised title is **“Evaluation of nutritional supplements prescribed, its associated cost and patients knowledge, attitude and practice towards nutraceuticals: A hospital based cross-sectional study in Kavrepalanchok, Nepal” [Addressed in Page No.1 Line No. 2-4]**

Abstract :

In methods, please mention the statistical test used during data analysis.
**Author’s Response:** Pearson Chi-square test (*x*^2^) for independence was used to find the association between socio-demographic variables and patients’ KAP (knowledge, attitude and practice) towards nutraceutical. One way ANOVA was used to compare the cost of nutraceutical among the different outpatient departments. **[Addressed in Page No. 3-4 Line No.41-44]**

Introduction:
Citation of the original source (instead of reference provided) is recommended particularly in paragraph 2. Author are advised not to provide the assumed hypothetical or contradictory statements regarding study intention and outcomes of study as laid down in paragraph 4 and 5 of the introduction section beside the rationale of the study needs be properly stated. The flow and smoothness in and during transition of paragraph need to be improved.

**Author’s Response:** We have revised the introduction section according to the comments of the reviewers.

Citation of the original source (instead of reference provided) **[Addressed in Page No. 6 Line No.91-96]**

*Ref.3: Bagchi D. Nutraceutical and functional food regulations in the United States and around the world: Elsevier; 2014.*

*Ref.4: Nutraceutical Market Size, Share & Trends Analysis Report By Product (Dietary Supplements, Functional Foods, Functional Beverages), By Region, And Segment Forecasts, 2020 - 2027 [cited 2021 February 6]. Available from:* [*https://www.grandviewresearch.com/industry-analysis/nutraceuticals-market*](https://www.grandviewresearch.com/industry-analysis/nutraceuticals-market)*.*

Paragraph 4 and 5**[Addressed in Page No. 9-10 Line No 170-181]**

Rationale of the study **[Addressed in Page No.8- 9 Line No 134-155]**

I also wish to also see a literature body related to advantages/ disadvantages associated with chaotic use of nutraceuticals, factors influencing consumer’s/prescribers attitude regarding nutraceuticals, marketing and regulatory facts regarding the sale and distribution of Nutraceutical particularly in content to Nepal all in one to two paragraph within the acceptable word count limit.

**Author’s Response: [Addressed in Page No. 7-8 Line No.123-141]**

*Although adverse event (AE) reporting for medicines is compulsory, very few countries have similar regulation for dietary supplements. In US Congress passed the Dietary Supplement and Nonprescription Drug Consumer Protection Act in 2006. Between 2008 and 2011, the FDA received over 4300 total AE reports from the industry, but before enacting the act it used to be voluntary with many reports from other than the industry [11]. The efficacy of fish oils (e.g., cod liver oil) in the diet has been demonstrated in several clinical trials, animal feeding experiments and* in vitro *models that mimic cartilage destruction in arthritic disease. Also, there is some evidence for beneficial effects of other nutraceuticals, such as green tea, herbal extracts, chondroitin sulphate and glucosamine [12]. However, many dietary supplements lack scientific evidence for or against the use, but they continue to be widely ingested throughout the world [13].*

*In Nepal, Dietary Supplement Guideline has been published by the Ministry of Agriculture and Livestock Development, regulating the nutraceuticals. However, unwarranted prescribing of nutraceuticals by physicians for financial benefits from the manufacturers has been reported continuously. Department of drug administration (DDA) has been attempting to stop such practices, which put the patients at the high economic burden and possible health hazard. Risky behaviours like the purchase of unauthorized herbal formulations and nutraceuticals from street vendors are prevalent in Nepal, which must be brought to a complete halt [14].*

Methods:
Inclusion criteria: you have mentioned “those who were prescribed nutraceuticals” will be enrolled in study. Though the inclusion is appropriate for evaluating of prescribing pattern. However, this can strongly introduce participant *bias and/or response bias for assessing KAP of nutraceutical.*

**Author’s Response:**

A limitation of this study is that we have only included the patients who were prescribed with nutraceuticals and this can lead to participant bias and/or response bias for assessing KAP of nutraceutical.

**[Addressed in Page No. 58 Line No. 835-837]**

Exclusion criteria: what about the geriatric and/or mentally retarded patients?

**Author’s Response:** The geriatric patients were not excluded. However, mentally disabled patients were excluded as they would not have answered our designed questions on nutraceutical KAP. In the exclusion criteria, we have now added the mentally disabled patients.**[Addressed in Page No. 11 Line No.207]**

Sampling techniques: Author have mentioned “stratified random sampling” in abstract whereas stratified purposive sampling is stated in methods section. Author is advice to reconcile the contradict section. Please mention how much sample size is taken from each departments with justification.

**Author’s Response: The sampling technique used was stratified purposive sampling. [Addressed in Page No. 3 Line No. 36-38]**

**How much sample size is taken from each departments with justification. [Addressed in Page No. 12 Line No. 218-223]**

*“The hospital has mainly 5 outpatient clinics; medicine, orthopedic, gynaecology, surgery and pediatrics. Since we excluded pediatrics, data collection from 4 clinics were performed. The total calculated sample size was 378. However, we took a sample of 400 patients, and since we had 4 departments, we took 100 patients from each of the 4 departments for proper distribution of the sample in strata.”*

Data collection tools and techniques:
Author is advised to cite the references article reviewed or undertaken while designing the tools/questionnaire of the study. Please state the mean score value for adequate, moderately adequate and inadequate knowledge, similarly for positive, moderately positive and negative attitude.

**Author’s Response:**
We have cited the reference articles reviewed while designing the study's questionnaire in the manuscript **[Addressed in Page No 13 , Line 253-254].**

- Gosavi S, Subramanian M, Reddy R, Shet BL. A study of prescription pattern of neutraceuticals, knowledge of the patients and cost in a tertiary care hospital. Journal of Clinical and Diagnostic Research: JCDR. 2016;10(4):FC01.
- Alhomoud FK, Basil M, Bondarev A. Knowledge, attitudes and practices (KAP) relating to dietary supplements among health sciences and non-health sciences students in one of the universities of United Arab Emirates (UAE). Journal of Clinical and Diagnostic Research: JCDR. 2016;10(9):JC05.
- Teoh SL, Ngorsuraches S, Lai NM, Bangpan M, Chaiyakunapruk N. Factors affecting consumers' decisions on the use of nutraceuticals: a systematic review. Int J Food Sci Nutr. 2019;70(4):491-512. Epub 2019/01/13. doi: 10.1080/09637486.2018.1538326. PubMed PMID: 30634867.

*Please state the mean score value for adequate, moderately adequate and inadequate knowledge, similarly for positive, moderately positive and negative attitude*.

**[Addressed in Page No 15-16, Line 299-305].**

Following are the mean score values for knowledge:

- Inadequate knowledge (<0.5)
- Moderately adequate knowledge (0.5-0.749)
- Adequate knowledge (0.5-1)
- Following are the mean score values for attitude:
- Negative attitude: <3
- Moderately positive attitude: 3-3.99
- Positive attitude: 4-5

Prescribing pattern categories of nutraceutical which you have laid down is not clear. Is there any appropriate references followed to classify nutraceutical? In my understanding. There are marginal differences between the nutraceutical (which are generally understood for food derived dietary supplements) and pharmaceutical supplements (which actually contains pharmaceutically active ingredients, properly dose regulated and quality controlled e.g. specific active vitamins supplement, folic acid, calcium and iron tablets). While both are nutritional health supplements, differentiation is important for better clarity (as pharmaceutical single or multiple doses form supplements are widely used /and are important in medical therapy while nutraceutical are often comes a dietary supplements and are not necessarily regulated and doesn’t require prescription, particularly in country like Nepal). Therefore, proper characterization of the types of nutraceutical needs to be done as per study aim else the readers may misinterpret the findings.
**Author’s Response:** Nutraceuticals or functional foods can be classified on the basis of their natural sources, pharmacological conditions, or as per chemical constitution of the products.

1. On the basis of natural source, it can be classified as the products obtained from plants, animals, minerals, or microbial sources.
2. Nutraceuticals as per the chemical groupings.

*Category of Nutraceutical*

- Substances with established nutritional functions, such as vitamins, minerals, amino acids, and fatty acids–Nutrients.
- Herbs or botanical products as concentrates or extracts–Herbals.
- Reagents derived from other sources (e.g., pyruvate, chondroitin sulfate, steroid hormone precursors) serving specific functions, such as sports nutrition, weight-loss supplements, conventional fortified foods, and meal replacements–Dietary supplements.

Some of the most common ways of classifying nutraceuticals can be based on food sources, mechanism of action, chemical nature, etc. The food sources used as nutraceuticals are all natural and can be categorized as

1. Dietary Fiber.
2. Probiotics.
3. Prebiotics.
4. Polyunsaturated fatty acids.
5. Antioxidant vitamins.
6. Polyphenols.
7. Spices

The category above has been extracted from the article which can be accessed in the following link <https://www.ncbi.nlm.nih.gov/pmc/articles/PMC3645360/#!po=17.8571>

We have also followed the classification of nutraceutical based on the article by Gosavi et al. where the prescription pattern categories of nutraceutical have been depicted as vitamins, minerals, proteins and probiotics. Mentioned below is the link to the referred article.

<https://www.ncbi.nlm.nih.gov/pmc/articles/PMC4866122/>

Data analysis and Results:
Prescribing pattern of Nutraceuticals:
You have mention in inclusion criteria as “Patients who were prescribed nutraceutical” are enrolled in study. The first line reads 80 % of patients are using/prescribed nutraceuticals? Please clarify.

**Author’s Response:** Eighty percent of patients were using/prescribed nutraceuticals refers to the percentage of patients who were already using nutraceutical in their daily life before the study was conducted due to the various reasons.

In the inclusion criteria, we have mentioned "Patients who were prescribed nutraceutical" which refers to the patients who have been prescribed nutraceutical either on the day of hospital visit or who were using nutraceutical from earlier time. **[Addressed in Page No.11 Line No.199-201]**

Table 2. Kindly find comments in methods section regarding categorization of nutraceuticals. I don’t think as per your study aim, there is any significance of tabulating the nutritional supplements categories as per the use by departments.
Tables could be made more concise and reader friendly, for e.g presenting the gender wise distribution of knowledge, attitude related table (table 3 and 8) in a single table, and similarly association related table (table 4 and 6) in another table, **while illustrating the significant results in text.**

**Author’s Response: [Addressed in Page No. 20 Line No.372-373]**

**Merging table 3 and 8 [Addressed in Page No. 23-25Line No.415-416]**

**Merging table 4 and 6 [Addressed in Page No. 33-35Line No.498-499]**

Data analysis remarks: you have used chi square test to determine the relationship between socio-demographic groups and KA level ( many of those consisting with 4/5*3 consistency table). Analysis with chi square to determine relationship in such cases may not be appropriate, and in many instances requires post hoc test for chi square for identifying where the significant association lies (if any) .

**Author’s Response: [Addressed in Page No. 33 Line No.483-490]**

Post-hoc test for chi-square was carried out for age and level of attitude (p=0.00).

|  | Attitude Level (Calculated P value) | | |
| --- | --- | --- | --- |
| Age (Years) | Negative attitude | Moderate Positive attitude | Positive attitude |
| 15-29 | 0.3 | 0.8 | 0.2 |
| 30-44 | 1.0 | 0.000 | 0.000 |
| 45-49 | 0.04 | 0.07 | 0.001 |
| >60 | 0.002 | 0.000 | 0.003 |

P value for post-hoc test was (0.05/12) = 0.004

Based on a post-hoc test of chi-square, association between age group 30-44 years and moderate positive attitude and positive attitude was found to be highly significant (p=0.000). Similarly, association between age group 45-49 years and positive attitude was also found to be significant (p=0.001). Furthermore, association between age group above 60 years and negative attitude, moderate positive attitude and positive attitude was found to be significant (p=0.002, p=0.000, p=0.003) respectively

Alternatively, distributing relevant socio-demographic variables (such as age, gender, literacy level, Marital status, employment status etc) wisely into binary group and then getting it compared with frequency score (using chi square) or with the mean/median score of Knowledge and attitude (e.g. using t test, Anova, Mann-whitney, KW test for means which ever suitable) may be more appropriate.

**Author’s Response:** We have addressed the comment on data analysis by carrying out the post-hoc test of chi-square **[Addressed in Page No. 33 Line No.483-490]**

Besides, determining the influence of predictor variables like age, literacy status, employment status etc and/or their co-relation for KAP will further strengthen the findings?

**Author’s Response:** Thank you for the recommendation. This has been put under the Strength and limitations section. We will consider this recommendation in future studies. **[Addressed in Page No.58 Line No.838-839]**

Table 7. Since it’s the patient’s perceived response to nutraceutical been prescribed, I don’t see any rationale for presenting it in a distributive form with respect to OPD clinics.

**Author’s Response:** Thank you for the comment. Table 7. has been modified as per the comment. **[Addressed in Page No.39-40 Line No.528-529]**

Table 9. Finding of Post hoc test for ANOVA can be explained in text and need not necessarily be presented in table.

**Author’s Response: Comment addressed in Table 9.[Addressed in Page No. 45-46 Line No. 568-570]**
Discussion:
Discussion needs refinements.
Strength and implication can be provided after the conclusion instead of first paragraph.
Some of the important background information are being highlighted in discussion (can be rearrange to Introduction section)

Author’s Response: Strength and implication are provided in “Strength and Limitation” section after the conclusion instead of first paragraph. **[Addressed in Page No.58 Line No.829-832]**

Some of the important background information are being highlighted in discussion (can be rearrange to Introduction section)

*In Nepal, Dietary Supplement Guideline has been published by the Ministry of Agriculture and Livestock Development, regulating the nutraceuticals. However, unwarranted prescribing of nutraceuticals by physicians for financial benefits from the manufacturers has been reported continuously. Department of drug administration (DDA) has been attempting to stop such practices, which put the patients at the high economic burden and possible health hazard. Risky behaviours like the purchase of unauthorized herbal formulations and nutraceuticals from street vendors are prevalent in Nepal, which must be brought to a complete halt.*

**[Addressed in Page No.8 Line No.134-141]**

The transition of paragraph needs to be properly arranged as per the order of finding of the result. Author are advised to compared their findings to more of the relevant/similar research literature of other developed and developing countries including that of South-Asian countries in terms of nutraceutical use and patients KAP.

**Author’s Response:** The transition of paragraph has been arranged as per the order of finding of the result.

Addition of literatures: **[Addressed in Page No. 48 Line No. 624-626; Page No. 52 Line No 697-700]**

Conclusion: Conclusion should be based only as per the finding of your study.

**Author’s Response: Conclusion has been revised. [Addressed in Page No. 57 Line No.816-824]**

Additional suggestion and recommendation could be made separately in recommendation section.
**Author’s Response:**

**Recommendation section has been added as per the reviewers' suggestion. [Addressed in Page No. 58 Line No.842-845]**

General comments: The article requires improvements in English, possible proof reading by native English speaker or professional expert/ services is recommended.

**Author’s Response:** We have done improvement in English.

Reviewer #2:

Overall impression, the language and formatting were poorly done.

**Author’s Response:** We have revised the manuscript thoroughly in terms of language and formatting.

The major concern about the paper is the methodological aspect, especially the sampling population and the content of the questionnaire. The methods despite not clearly described do not seem to me to have reached a scientific enough level.
**Author’s Response:** The methodology section has been revised and the details that had not been put in the initial draft of manuscript has been added.

Some other specific comments:

Abstract
1) Not convinced with the implication of use of the research that has been described in the abstract's conclusion.

**Author’s Response:** We have the revised the abstract’s conclusion. **[Addressed in Page No.5 Line No. 66-71]**

2) Please use a more appropriate language for "Vitamins were prescribed maximum in medicine ".
**Author’s Response:** Vitamins were the most widely prescribed nutraceuticalin our study, followed by minerals, enzymes and herbal which is in agreement with the study conducted in India among the health science students. [**Addressed in Page No. 48 Line No.622-624]**

Introduction
1) Line 82-87 describes about the use of nutraceuticals for malnutrition using WHO's statistics. I am not agreeable to the purpose of the use of nutraceuticals in your context. I believe the participants here were taking nutraceuticals for "dietary supplementation" instead of "malnutrition". Malnutrition is normally for those in critical environment.

**Author’s Response:
Thank you for the comment. Associated information and statistics with malnutrition has been removed from the introduction section [Addressed in Page No.9 Line No. 162-170]**

Method
1) Line 119-120 "The total population of a patient visiting OPD was 22500 (with an average 250 per day) in three-month duration according to hospital census." should be in results instead of methods or after the descriptions of sampling method.

**Author’s Response:**

In the sample size calculation formula, N stands for total number patient visited in three-month duration as per censes. Hence the information of total population of patients visiting OPD in three-month duration as per hospital census was added in sampling method section.

n=N.Zα/2². P (1-P)/ [(N-1) e² + Zα/2². P (1-P)]

Where, n = minimum sample size of the study subject

N = total number patient visited in three-month duration as per censes

Z = standard normal distribution curve /value at α/2 for the 95% confidence interval (1.96)

P = proportion of population with KAP of nutraceutical among respondents (50%)

e = the margin of error (0.05)

***N = total number patient visited in three month duration as per censes***

2) Line 124 - 127 - "A questionnaire was designed after reviewing the previous similar studies with some modifications. Structured questions were used for the collection of data, and the prescriptions and bills were scrutinized" - I am not convinced about the appropriateness of the content of the developed questionnaires.

**Author’s Response:** Revision as per the comment has been done under "Data collection tool and technique" section. **[Addressed in Page No. 13-14 Line No. 253-260]**

3) There were no referred versions to be seen. No experts to examine the content of questionnaire?

**Author’s Response:**

References for the published literatures that were referred to in the process of developing the questionnaire has been added "Data collection tool and technique" section **[Addressed in Page No. 13-14 Line No. 253-260]**

3) Line 128 - participants were randomly selected? How? It was not described

**Author’s Response:** The participants were selected purposively. Stratified purposive sampling technique was used. Needful correction has been made in "Sampling method and technique" section. **[Addressed in Page No.12 Line No. 218-223]**

4) Line 149-150 - How the calculation of "the average percentage of costs spent on nutraceutical 150 alone compared to the total prescribed drugs were measured following OPD." is performed, it is not clearly described.

**Author’s Response:**
The cost for the individual category of nutraceutical and other medications were noted. The cost of nutraceutical per prescription was calculated for each patient.

Average nutraceutical cost % on prescription = (Total cost of nutraceutical/Total prescription cost) *100% **[Addressed in Page No.17 Line No. 321-326]**

Results
1) 201 - "Do you know what nutraceutical are?" Does not seem to be accurately testing the content of the "knowledge" of participants. "Nutraceuticals" itself is a very jargonish term even researcher of healthcare providers may not be aware of.

**Author’s Response:** Thank you for the comment. We had used the term nutraceutical because this is the common term used among healthcare providers in Nepal. When there was confusion the data collector assisted by explaining to confused ones. Further, we will consider your valuable suggestion in future studies related to nutraceuticals.
