# Supplementary material for: Evaluation of nutritional supplements prescribed, its associated cost and patients knowledge, attitude and practice towards nutraceuticals: A hospital based cross-sectional study in Kavrepalanchok, Nepal
Source: PLoS One. 2021 Jun 4;16(6):e0252538. doi: 10.1371/journal.pone.0252538 (PMC8177421; doi:10.1371/journal.pone.0252538)
Supplement: Attachment — Submitted filename: Response-to-reviewers (1).docx [file pone.0252538.s005.docx]

16^th^ April, 2021

Response to reviewers

Dear Editor,

Thank you very much for providing us the comments on improving the manuscript level as per the Plos One standard. We believe that the revised version of the manuscript has been improved as a result of the reviewers’ comments and feedback and the re-revised version has been successful in meeting the Plos One standard, particularly improving the English level (as per the additional editor comments) and the editor comments on cross-checking the references to respond the retracted article in proper way (if any).

Please find the revised manuscript “Evaluation of nutritional supplements prescribed, its associated cost and patients knowledge, attitude and practice towards Nutraceuticals: A hospital based cross-sectional study in Kavrepalanchowk, Nepal”.

As per the suggestion from editor, we have used the fluent English writer to have a look at our manuscript and thus we believe that the quality of the manuscript has been improved. We also have used single level of track changes mode-on for not adding difficulty to editor to assess the changes made. At some places, the manuscript has been edited for brevity.

Best wishes

Sweta Shrestha
