# Supplementary material for: Evaluation of nutritional supplements prescribed, its associated cost and patients knowledge, attitude and practice towards nutraceuticals: A hospital based cross-sectional study in Kavrepalanchok, Nepal
Source: PLoS One. 2021 Jun 4;16(6):e0252538. doi: 10.1371/journal.pone.0252538 (PMC8177421; doi:10.1371/journal.pone.0252538)
Supplement: Attachment — Submitted filename: Response-to-reviewers (1).docx [file pone.0252538.s006.docx]

Date: 7^th^ May 2021

Jenny Wilkinson, PhD

Academic Editor

PLOS ONE

Subject: Response to Editor and submission of the revised version of the manuscript

Dear editor,

Thank you very much for providing us with the comments on improving the manuscript level as per the Plos One standard. We have revised manuscript titled **“Evaluation of nutritional supplements prescribed, its associated cost and patients knowledge, attitude and practice towards Nutraceuticals: A hospital based cross-sectional study in Kavrepalanchowk, Nepal**”, **Manuscript ID: PONE-D-20-36161R2** and submitting you the revised manuscript in both track changes and clean version.

We have mentioned a point-to-point response below (in the blue colour font) to the comments by the editor.

Yours Sincerely,

Sweta Shrestha on behalf of the study team.

**Comments:**

Journal Requirements: Please review your reference list to ensure that it is complete and correct. If you have cited papers that have been retracted, please include the rationale for doing so in the manuscript text, or remove these references and replace them with relevant current references. Any changes to the reference list should be mentioned in the rebuttal letter that accompanies your revised manuscript. If you need to cite a retracted article, indicate the article’s retracted status in the References list and also include a citation and full reference for the retraction notice.

**Response:** We believe that the revised version of the manuscript has been improved because of the reviewers’ comments and feedback, and the re-revised version has been successful in meeting the Plos One standard, particularly improving the English level (as per the additional editor comments) and the editor comments on cross-checking the references to respond the retracted article properly (if any).

Additional Editor Comments (if provided):

Thank you for your revisions, while the content related issues have been addressed there are still many grammatical and language issues. For example, the sentence “Many schools and universities started switching from traditional classroom teaching to virtual education methods” is repeated at the top of page 3, ‘et al’ is incorrectly shown with full stops after both et and al and author initials included in in-text citations, etc. I strong advise seeking assistance from a native English writer experience in academic writing or professional editing service.

**Response:** The sentence “Many schools and universities started switching from traditional classroom teaching to virtual education methods” you mentioned is not mentioned in our manuscript. We believe that there is some confusion. Please kindly verify it once. As per the suggestion from the editor, we have used a fluent English writer to look at our manuscript, and thus we believe that the quality of the manuscript has been improved. We have also used the single level of track changes mode to make it easier for the editor to assess the changes made. At some places, the manuscript has been edited for brevity.
